# Code vulnerability detection based on augmented program dependency graph and optimized CodeBERT

**DOI:** 10.1038/s41598-025-23029-4

**Published:** 2025-11-10

**Authors:** Zhengbin Zou, Tao Jiang, Yizheng Wang, Tiancheng Xue, Nan Zhang, Jie Luan

**Affiliations:** School of Mathematics and Computer Science, Yunnan Minzu University, Yuehua Street, Kunming, 650504 Yunnan China

**Keywords:** Vulnerability detection, Natural language processing, Program dependency graph, Pretrained model, Software, Computer science

## Abstract

The increasing complexity of software systems has rendered code vulnerability detection a critical aspect of software security. While deep learning-based approaches have advanced this field, challenges such as coarse-grained function-level detection, scalability limitations, and constrained accuracy persist. Although slice-level detection effectively reduces noise, it frequently sacrifices essential syntactic and semantic information, which undermines vulnerability representation, elevates false positive rates, and ultimately limits practical applicability. To address these challenges, this paper proposes a code vulnerability detection method based on an augmented program dependency graph and optimized CodeBERT. The method augments the traditional program dependency graph by extending its structure to capture richer semantic and structural information in the code. Furthermore, it employs the Code Bidirectional Encoder Representations from Transformers (CodeBERT) pretrained model for extracting code embedding features. Additionally, a hybrid loss function optimization strategy tailored for CodeBERT is proposed to address the long-tail distribution characteristics of code vulnerability detection. The experimental results demonstrate that, compared to other state-of-the-art classical methods, the detection accuracy and F1 score on synthetic and real-world datasets have been improved by an average of up to 8.34% and 29.71%, respectively.

## Introduction

In the current digital era, software applications have become integral to daily work and life. However, due to developer oversights, design flaws, improper implementation, and insufficient testing, numerous security vulnerabilities frequently emerge in software applications. These issues have rendered code vulnerability detection a persistent and critical challenge in the domain of software security^1–3^.Consequently, enhancing software security defenses and advancing vulnerability detection methods are of paramount importance.

Despite the widespread adoption of traditional vulnerability detection methodologies—including static analysis, dynamic testing, and machine learning techniques—these approaches exhibit significant limitations. Static analysis is susceptible to code complexity and contextual dependencies, often resulting in false negatives or false positives. Dynamic testing, reliant on test case coverage, frequently fails to detect vulnerabilities obscured by untested execution paths. Conventional machine learning methods face challenges such as prohibitive manual annotation costs, inefficient training processes, and insufficient data availability^4,5^. Moreover, as code complexity and vulnerability diversity escalate, these traditional methods increasingly struggle to meet practical demands for accuracy, reliability, and adaptability.

Recent years have witnessed growing interest in deep learning-based vulnerability detection due to its capacity for automated feature learning. However, existing deep learning applications in this domain confront critical challenges. First, prevailing methods often disregard code structural properties, over-relying on linear representations of source code, which compromises accuracy in capturing vulnerability features^6–8^. Second, deep learning models demonstrate pronounced susceptibility to overfitting when handling intricate vulnerabilities and exhibit limited efficacy in addressing both the heterogeneity of vulnerability types and class imbalance issues, thereby perpetuating false negatives and positive^9–11^.

In response to the aforementioned challenges, this paper proposes a code vulnerability detection method based on augmented program dependency graph (AUG-PDG) and optimized CodeBERT. Firstly, an AUG-PDG is constructed by adding five additional edge types. Secondly, the subgraph of the AUG-PDG is obtained using program slicing technology, and the source code is sliced and normalized to reduce the interference of redundant information in the samples. Thirdly, the CodeBERT pretrained model is used for slice embedding and feature extraction, thereby obtaining its vector representation to preserve the structural information of the source code and vulnerability features. Finally, the class imbalance problem and optimizes the model loss by introducing a hybrid loss function optimization strategy tailored to CodeBERT.

The following are the principal contributions that this paper offers:

- This paper proposes a code vulnerability detection method, AugSliceVul, based on an AUG-PDG and optimized CodeBERT. This method leverages pretrained models to capture the contextual information of slices, thus facilitating the understanding of semantic information in code slices for vulnerability detection.
- Constructs a program representation method based on the AUG-PDG and employs slicing techniques to mitigate interference from irrelevant statement nodes in the program dependency graph (PDG) structure. Unlike conventional PDG structures, the AUG-PDG incorporates a more comprehensive representation of semantic information by accounting for both fundamental control and data dependencies, as well as complex interactions among diverse program elements.
- The program slicing algorithm is improved by incorporating a dynamic root node selection strategy and a bidirectional dependency traversal mechanism. Targeting four types of high-risk vulnerabilities—such as pointer operations and array out-of-bounds—a forward tracking of variable usage chains and a backward tracing of definition sources are employed to generate logically complete vulnerability slices, thereby reducing interference from redundant code.
- A hybrid loss function optimization strategy for CodeBERT is proposed, which integrates weighted cross-entropy loss and focal loss to optimize the loss function of the CodeBERT-based classification model. This approach is designed to address the long-tail distribution characteristic of code vulnerability detection and enhance the model’s capability in identifying hard examples, such as cross-scope vulnerabilities.
- This paper performed experiments extensive in scope on synthetic and real-world datasets. On the synthetic dataset, the proposed method achieved an average accuracy of 99.84%, precision of 99.26%, recall of 99.60%, and an F1 score of 99.41%. On the real-world dataset, it achieved an average accuracy of 98.01%, precision of 84.34%, recall of 95.58%, and an F1 score of 89.58%. Experimental evaluations indicate that the implemented approach identified vulnerabilities across both artificially generated and real-world datasets.

The organization of this paper is as follows. “Related works” presents the related work; “Methodology” details the design and implementation of the proposed method; “Experiment” reports the experimental setup and compares the results with mainstream vulnerability detection methods; finally, the conclusion is provided in the last section.

## Related works

In the domain of source code vulnerability detection, researchers predominantly utilize three main approaches: metric-based, machine learning-based, and deep learning-based detection. These methods, while distinct in their methodologies, are not entirely independent and often exhibit complementary characteristics.

Vulnerability detection based on code metrics focuses on evaluating multiple quantitative dimensions of code. The method by Shin et al.^12^ignores vulnerability context, leading to missed detections. Chowdhury et al.^13^cannot adapt to the complexity of different code types. Zhou et al.^14^ rely on textual data, making it difficult to fully account for code structure. Zagane et al.^15^evaluate both the macro and micro structures of code, but incur high computational overhead. In summary, existing code-metric-based vulnerability detection methods suffer from poor adaptability to code context and structural characteristics, as well as high computational overhead in large-scale codebases.

Machine learning-based vulnerability detection methods typically rely on domain experts to manually select features. Hovsepyan et al.^16^failed to effectively integrate code structure, affecting accuracy. Yang et al.^17^shows poor adaptability and scalability when facing different programming languages and complex projects. Ahmadi et al.^18^ depend on predefined feature selection, limiting their ability to capture complex vulnerabilities. In summary, existing machine learning-based vulnerability detection methods have limitations in feature selection, adaptability, and the ability to capture complex vulnerabilities.

Deep learning–based detection methods have replaced manual feature definitions with automatic feature extraction, stimulating research interest in both academia and industry. Li et al.^7^ proposed VulDeePecker, which uses Bi-LSTM to capture contextual information of code but has limited ability to handle long-range dependencies. Zhou et al.^8^Devign incurs high computational overhead in large-scale codebases, exhibits poor adaptability, and achieves low accuracy. Li et al.^10^ proposed SySeVR, which loses a large amount of semantic information when capturing code structure, resulting in low accuracy. Wu et al.^11^ introduced VulCNN, which extracts features through a multi-layer convolutional neural network, but CNN’s overemphasis on local features limits the learning of global vulnerability patterns. Liu et al.^19^ proposed the Vul-LMGNN model for vulnerability detection based on graph structures, but it heavily relies on code structure and lacks the flexibility to handle different code contexts. Luo Leqi et al.^20^ transformed source code into approximate natural language and used the BERT model for automatic feature extraction, but excessive noise degraded performance and reduced efficiency. Zhang et al.^21^ proposed the Context Slicing method, combining GCN and BGRU to extract information, but this method may encounter challenges with insufficient feature representation in large-scale codebases. Cao Binghao et al.^22^ used Joern to generate and optimize CPG, vectorized it through word2vec, and then used GAT for vulnerability detection; however, this method demands high-quality graph representations and may overlook code’s semantic and syntactic information. Xiansheng Cao et al.^23^ used Bi-LSTM and GNN to learn features and designed a dual-attention mechanism, but the method has high computational complexity and may fail to fully understand potential vulnerabilities in code. Ji Youqing et al.^24^ used CodeBERT and Swin Transformer for vulnerability detection, but they may encounter performance bottlenecks when handling complex dynamic behavior. In conclusion, deep learning-based detection methods have significant limitations in feature extraction, code semantic loss, accuracy, computational overhead, and handling complex vulnerabilities.

In summary, traditional vulnerability detection methods possess their respective strengths but are limited in terms of accuracy and generalization capability. Although deep learning-based approaches can automatically extract features, current methods still have room for improvement in capturing the structural and semantic information of code, with certain limitations in feature extraction and accuracy. To address these issues, this paper introduces AugSliceVul, which integrates the AUG-PDG with pretrained models to augment the accuracy and reliability of vulnerability detection.

## Methodology

### Framework

**Fig. 1:**
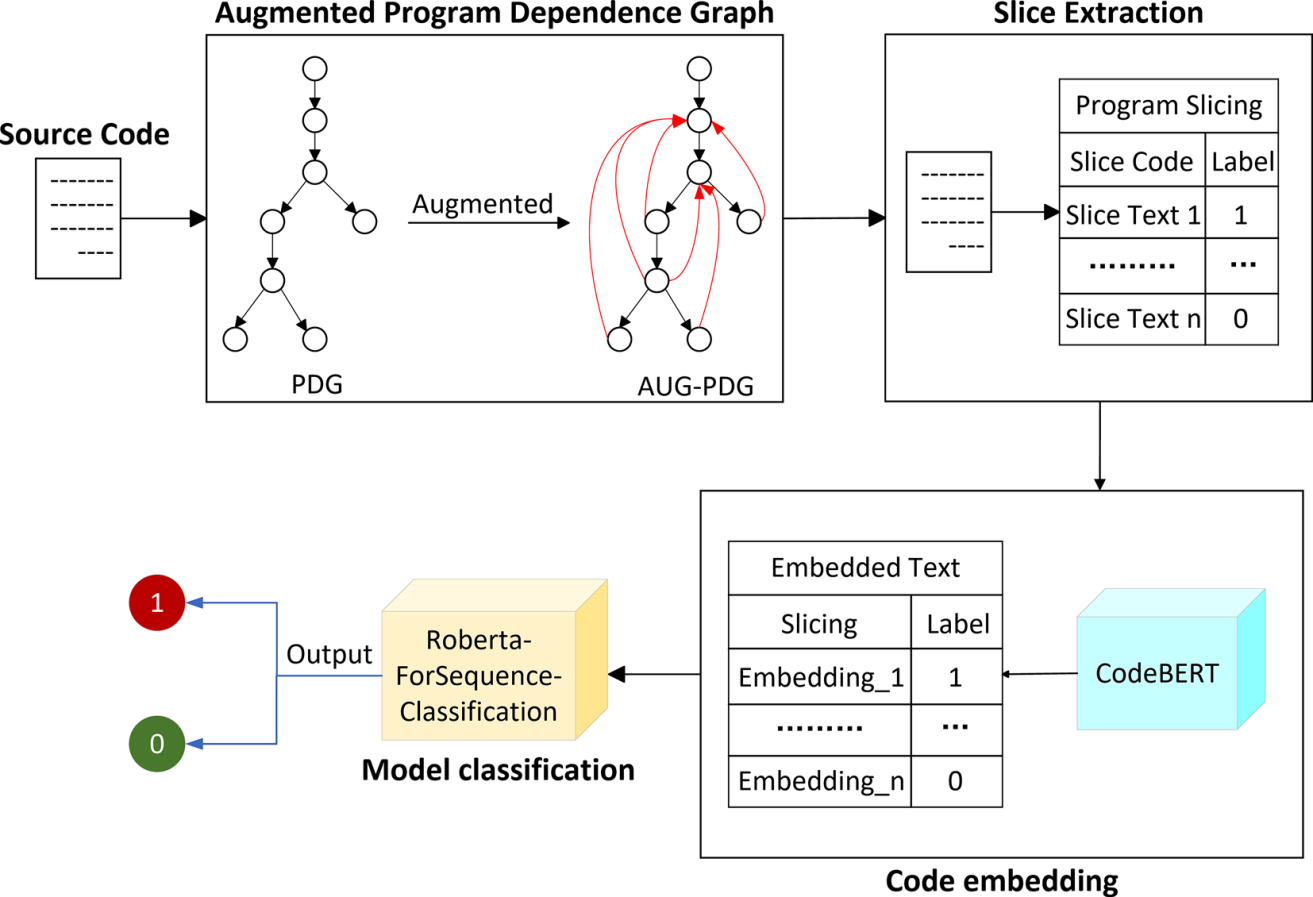
Overall framework.

This paper proposes and implements a code vulnerability detection method, AugSliceVul, based on an AUG-PDG and an optimized CodeBERT. As presented in Fig. 1, the overall architecture of the method is composed of four modules: augmented program dependency graph, slice extraction, code embedding, and model classification. The input is C/C++ source code files at the function-level granularity.

- Augmented program dependency graph module: the Joern^25^ tool is initially employed to generate a basic PDG. Subsequently, a PDG extension algorithm is utilized to add five additional edge types—UseDef, ExScopeGroup, VarEnd, VarScopeEnd, and ScopeEnd—enabling the AUG-PDG to capture more comprehensive program data and control flow information.
- Slice extraction module: program slicing is performed based on the constructed AUG-PDG. The extracted slices undergo data preprocessing, during which the slice code is paired with corresponding labels and normalized to ensure consistency and reduce noise.
- Code embedding module: the preprocessed program slices are input into the CodeBERT model for embedding. This process converts the slice code into two-dimensional tensor representations, preserving both structural and semantic features of the code.
- Model classification module: the vector representations generated in the code embedding module are utilized for classification. By analyzing these vectors, the model determines the presence or absence of vulnerabilities in the program slices. The final output provides a precise vulnerability assessment, indicating whether the given C/C++ code contains vulnerabilities.

The proposed method achieves vulnerability detection in C/C++ source code through the synergistic interaction of four modules: augmented program dependency graph, slice extraction, code embedding, and model classification.

### Augmented program dependency graph

The PDG is a graphical representation technique used to visualize data flow and control flow dependencies among program instructions^26^. This representation plays a critical role in advanced program analysis, performance optimization, and parallel processing tasks. By combining nodes and edges, the PDG provides an intuitive depiction of a program’s execution logic and data flow pathways. q In this paper, the Joern tool is employed to construct the PDG, which is subsequently optimized to enhance its representation capabilities. To capture richer syntactic, data, and control flow information, additional edge types are introduced into the original PDG, modeling complex syntactic and semantic relationships between nodes. These enhancements result in the construction of the AUG-PDG.

**Fig. 2:**
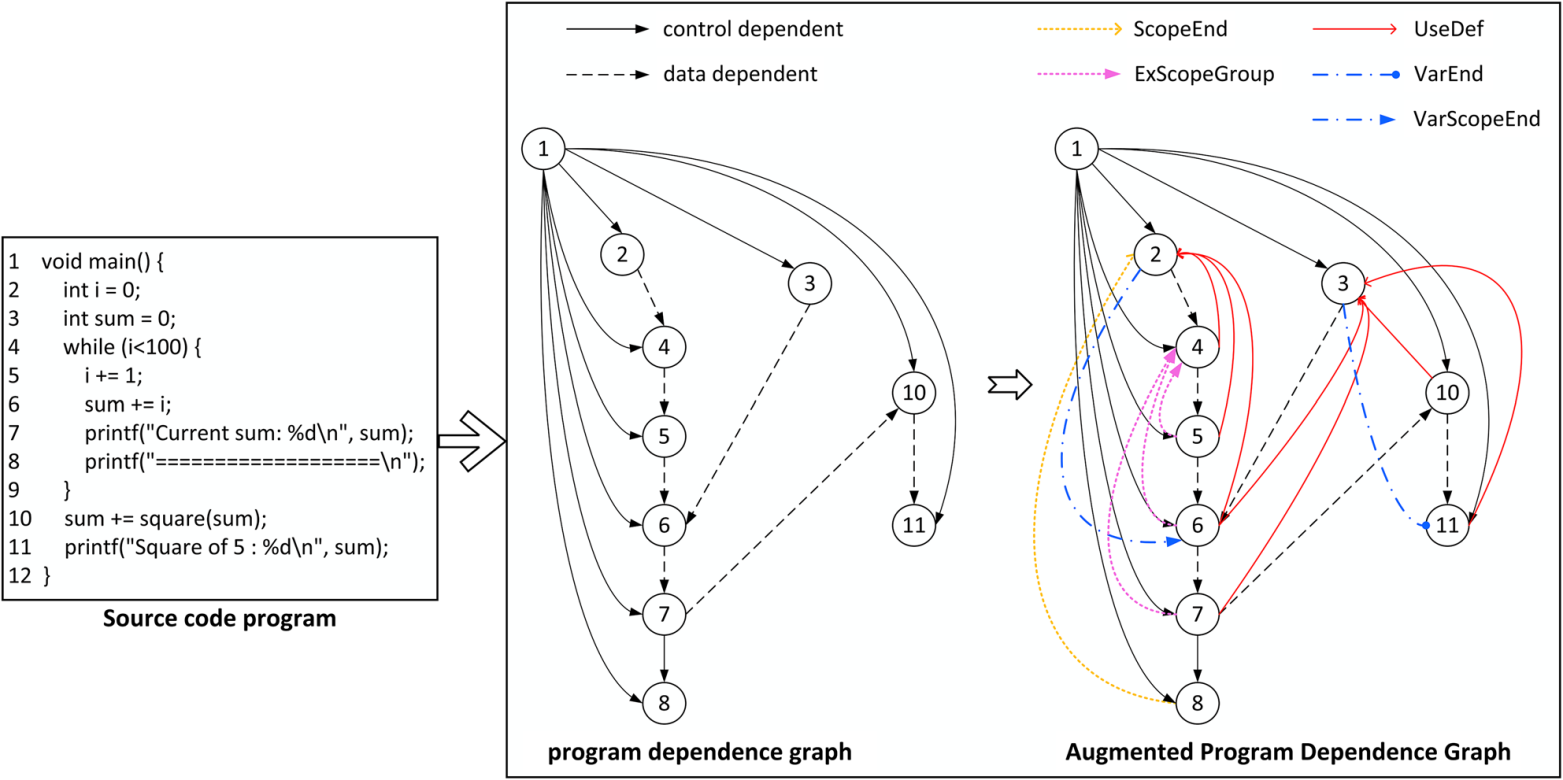
Transformation process for constructing AUG-PDG from source code.

This paper introduces five new edge types—UseDef, ExScopeGroup, VarEnd, VarScopeEnd, and ScopeEnd—to enhance the PDG. These edges are designed to better represent the syntactic and semantic intricacies of the code. The process of constructing the AUG-PDG from source code is illustrated in Fig. 2. In the AUG-PDG, nodes represent program elements (e.g., variables, expressions, and statements), while edges denote the relationships between these elements. In Fig. 2, the edges of the original PDG are classified into two categories:

- Data dependency edges, which represent the associations between data elements in the graph.
- Control dependency edges, which denote the control flow relationships among statements.

The specific descriptions of the newly added edges are detailed below.

- UseDef: represents data flow dependencies by capturing the relationship between the use of a variable and its origin, enhancing the PDG’s ability to analyze data flow origins. This directed edge forms the core structure of data flow analysis, enabling precise tracking of variable sources and dependency chains, thereby improving the accuracy of variable origin analysis.
- ExScopeGroup: captures the dependency relationships between variables within a scope and their associated conditional expressions, reflecting the interdependencies among variables in a specific code block. This edge enhances the PDG’s representation of internal variable interactions within a scope, providing a clearer semantic expression.
- ScopeEnd: denotes the logical endpoint of a scope structure, offering a distinct marker of scope closure within the PDG. This edge facilitates a more explicit hierarchical termination when analyzing complex nested structures, thereby clarifying the structural dependencies of scope boundaries.
- VarEnd and VarScopeEnd: represent the last usage positions of the same variable, refining the delineation of variable lifecycles. VarEnd ensures a unified terminal marker for each variable’s lifecycle, while VarScopeEnd focuses on survival boundaries within a scope. These directed edges provide finer-grained control over the management of local variables in complex structures, thereby enhancing the precision of scope-specific variable analysis.

### Slice extraction based on augmented program dependence graphs

In traditional program slicing methods, slice generation typically relies on control flow and data flow dependencies within the Program Dependence Graph (PDG). However, conventional PDGs have limitations in capturing critical semantic information such as variable scope and variable lifetime, which often results in slices containing redundant code, including out-of-scope variable operations or dereferencing of invalid pointers. These limitations increase the susceptibility of traditional methods to noise in complex vulnerability detection tasks. To address these issues, this paper adopts program slicing techniques to extract subgraphs from an augmented program dependence graph (AUG-PDG) as candidate vulnerability slices. Furthermore, the code slicing algorithm proposed in the SySeVR framework^10^ is modified. The specific modifications are as follows:

Initially, a dynamic root node selection strategy is introduced by incorporating the explicitly modeled variable lifetime (VarEnd and VarScopeEnd edges) and scope boundaries (ScopeEnd edges) in the AUG-PDG, aiming to cover four types of high-risk vulnerability patterns, such as pointer operations and array out-of-bounds access.Subsequently, a bidirectional dependency traversal mechanism is designed. From the forward perspective, the algorithm traces the subsequent usage chain of variables along UseDef edges and utilizes VarScopeEnd edges to identify the valid lifetime of variables within their scope, thereby avoiding the inclusion of out-of-scope operations. From the backward perspective, the algorithm traces the definition sources of variables and control conditions (e.g., unchecked user inputs), ensuring a complete representation of the vulnerability-triggering logic.Additionally, the semantic information in AUG-PDG is leveraged to normalize the slices (e.g., unifying variable names), thereby reducing interference caused by redundant code.

The slicing Algorithm 1 proposed in this paper takes a C/C++ program D and a slicing criterion S as inputs. Initially, a control flow graph (CFG) and a program dependence graph (PDG) are constructed from D, based on which an augmented program dependence graph (AUG-PDG) is further built. The AUG-PDG is traversed to record function call relationships. According to the slicing criterion S, four types of high-risk root nodes are dynamically selected to identify the root node set V. For each node in V, the algorithm performs forward traversal along variable usage chains and backward tracing to definition sources within the AUG-PDG, collecting all encountered nodes during the traversal to generate a list of program slices $N_s$.The extracted slice list $N_s$ is subsequently processed through data preprocessing to facilitate downstream analysis. The preprocessing step ensures a one-to-one correspondence between the sliced code and their associated labels, thereby simplifying subsequent tasks. In addition, code slices are normalized to achieve consistent formatting and representation, enhancing their applicability in advanced analysis and practical scenarios.

**Algorithm 1:**
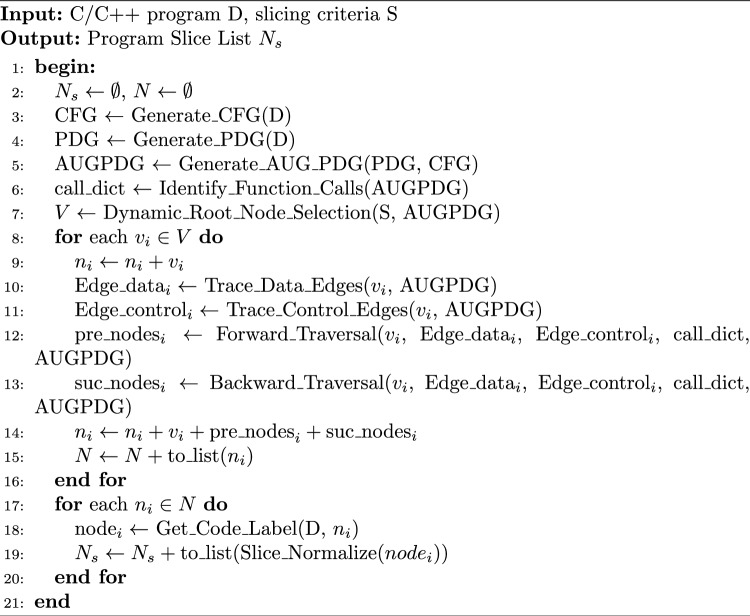
Program slicing algorithm.

### Code embedding based on the CodeBERT pretrained model

Code embedding converts program code into continuous vector representations, enabling the understanding of code semantics and logical relationships^27^. To effectively capture the intricacies of code, code embedding must address two critical considerations: (1) Vulnerable code contains critical elements like variable declarations and conditional judgments, which should be captured along with information from other critical points. (2) Code statements within a function are sequential and logically related, requiring embedding to preserve long-distance dependencies and maintain semantic accuracy.

This paper leverages CodeBERT to perform code embedding, utilizing its multi-layer Transformer encoder to produce semantic representations of source code. The multi-head self-attention mechanism lies at the heart of CodeBERT, which computes token-level relationships to capture variable dependencies and control flow information. This mechanism enables CodeBERT to focus on critical code components effectively, facilitating the extraction of features across diverse key regions^28,29^.

In order to capture the long-distance relationships within the code, positional encoding is incorporated into the token embeddings of the input layer. By adding positional encodings to the token vectors, the model gains the ability to better preserve structural and semantic features, capturing logical connections across distant code statements^29^. The operations are defined in Eqs. (1) and (2):

1 $$\begin{aligned} P E_{(p o s, 2 i)}=\sin \left( \frac{p o s}{10000^{\frac{2 i}{d_{\text{ model } }}}}\right) \end{aligned}$$

2 $$\begin{aligned} P E_{(p o s, 2 i+1)}=\cos \left( \frac{p o s}{10000^{\frac{2 i}{d_{\text{ model } }}}}\right) \end{aligned}$$

CodeBERT employs its multi-head attention mechanism to capture critical code elements, facilitating tasks such as code embedding and vulnerability detection. In security vulnerability detection scenarios, given the prevalence of logical dependencies and multi-level structures, ensuring a consistent length for the input sequence is of great significance. For long code fragments, the model truncates the input to preserve relevant information, while zero-padding is applied for shorter fragments to ensure uniformity. This preprocessing approach preserves the code’s overall structure and emphasizes key semantic features, enhancing CodeBERT’s performance in embedding and detection tasks.

### Vulnerability classification and detection based on a CodeBERT-oriented hybrid loss function optimization strategy

In code vulnerability detection tasks, severe class imbalance between positive and negative samples, along with the difficulty in identifying hard examples, poses significant challenges to model performance. Traditional cross-entropy loss is sensitive to class imbalance, while standalone focal loss may struggle to effectively distinguish critical vulnerability patterns from noise in complex code semantics. To address these issues, this paper proposes a hybrid loss function optimization strategy tailored to CodeBERT, which combines weighted cross-entropy loss and focal loss to dynamically balance sample weights and enhance the learning of hard examples.

In the model classification module, this paper employs the RobertaForSequenceClassification model to classify the embedding vectors of program slices and detect potential vulnerabilities in code snippets. The original model employs the standard cross-entropy loss function, expressed as:

3 $$\begin{aligned} L_{C E}=-\sum _{i=1}^N\left[ y_i \cdot \log \left( P_i\right) +\left( 1-y_i\right) \cdot \log \left( 1-P_i\right) \right] \end{aligned}$$

Let $P_i$ denote the true label of the i-th sample and $y_i$ represent its predicted label. The standard cross-entropy loss function performs well on balanced datasets; however, its performance significantly degrades when applied to imbalanced datasets, particularly in scenarios where rare vulnerability samples are vastly outnumbered by normal samples. This limitation arises because the standard cross-entropy loss function does not account for the differing importance of samples across classes.

To address this issue, a binary loss function combining Weighted Cross-Entropy Loss^30^ and Focal Loss^31^ is introduced during the training process to expand the model’s performance on rare vulnerability samples. The mathematical representation of the integrated loss framework is defined below:

4 $$\begin{aligned} L_{\text{ total } }=\lambda \cdot L_{W C E}+(1-\lambda ) \cdot L_{F L} \end{aligned}$$

Here, $\lambda$ is a tuning parameter that controls the contribution ratio of the Weighted Cross-Entropy Loss and Focal Loss to the overall loss. The Weighted Cross-Entropy Loss serves as the first part of the binary loss function, which addresses the class imbalance problem by assigning higher weights to minority class samples (vulnerability samples)^30^. The formulation of this loss function is as follows:

5 $$\begin{aligned} L_{W C E}=-\sum _{i=1}^N\left[ w_{p o s} \cdot y_i \cdot \log \left( P_i\right) +w_{n e g} \cdot \left( 1-y_i\right) \cdot \log \left( 1-P_i\right) \right] \end{aligned}$$

In this formulation, $w_{pos}$ and $w_{neg}$ represent the weights for the positive and negative classes, respectively, while $P_i$ denotes the true label of the i-th sample and $y_i$ represents its predicted label. By setting $w_{pos}$ > $w_{neg}$, the model can more effectively identify rare vulnerability samples.

The second component of the binary loss function is Focal Loss, which introduces a modulation factor to enable the model to focus more on hard-to-classify minority class samples^31^. Its formulation is as follows:

6 $$\begin{aligned} L_{F L}=-\sum _{i=1}^{N}\left[ \alpha \cdot \left( 1-P_{i}\right) ^{\gamma } \cdot y_{i} \cdot \log \left( P_{i}\right) +(1-\alpha ) \cdot P_{i}^{\gamma } \cdot \left( 1-y_{i}\right) \cdot \log \left( 1-P_{i}\right) \right] \end{aligned}$$

Here, $\alpha$ is a balancing factor that adjusts the contribution of positive and negative samples to the overall loss, while $\gamma$ is a tuning factor that reduces the loss for easily classified samples and increases it for hard-to-classify ones. This mechanism ensures that the model can effectively detect rare and complex vulnerability patterns.

The model utilizes a binary loss function that integrates Weighted Cross-Entropy Loss and Focal Loss for backpropagation and optimization. This loss function design effectively addresses the class imbalance problem, enhancing the model’s robustness in handling rare vulnerability samples. Weighted Cross-Entropy Loss ensures accurate classification of underrepresented classes by assigning higher weights to minority samples^30^, Focal Loss, on the other hand, reduces the impact of easily classified samples, enabling the model to concentrate on more challenging, hard-to-classify cases^31^. During the fine-tuning process, the model updates its weight parameters through the backpropagation algorithm, allowing it to adapt to the unique characteristics of code slices and improve its predictive performance.

**Fig. 3:**
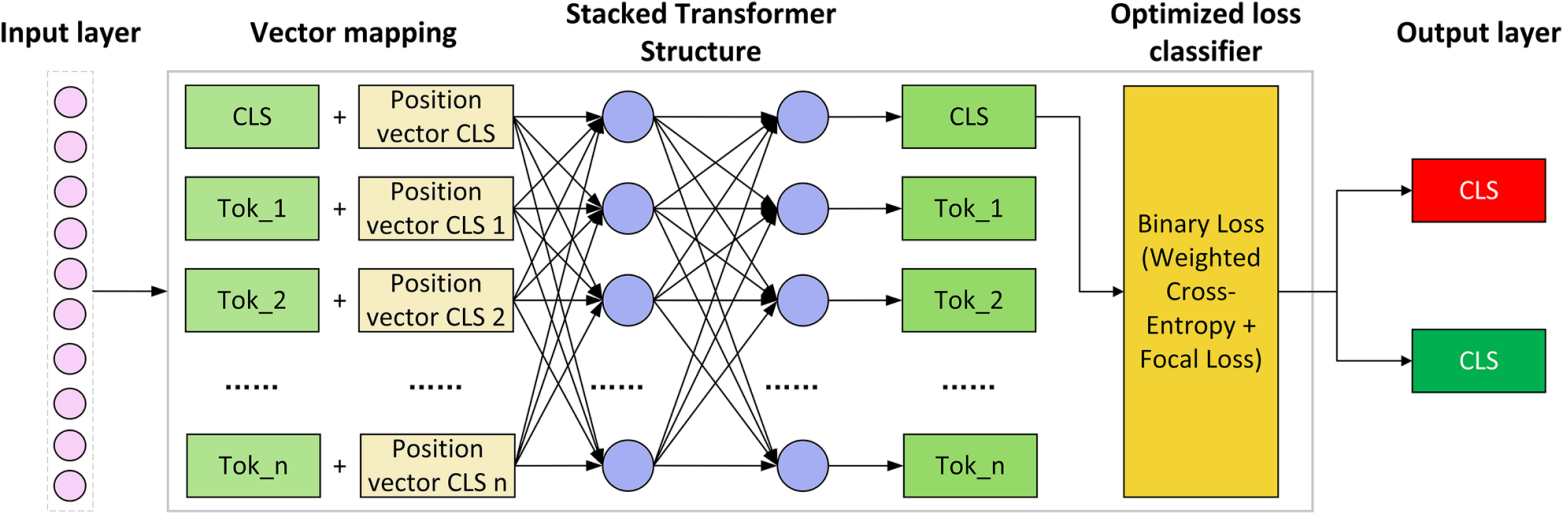
Structure of the vulnerability classification method based on optimized loss function.

Figure 3 illustrates the structure of a vulnerability classification method based on an optimized loss function. The input layer receives a sequence of tokens, and the processed token sequence is combined with positional embeddings to form an input representation that incorporates contextual information. A specific token ([CLS]) is appended to the beginning of the token sequence. These vectors are then fed into a stacked Transformer architecture for feature extraction. The model employs an optimized loss classifier (Weighted Cross-Entropy + Focal Loss) to enhance its ability to identify minority vulnerability samples. The final output is a binary classification result indicating whether the code fragment contains a vulnerability.

## Experiment

### Experiment data

The synthetic dataset used in this paper is sourced from SySeVR^10^, which is based on the SARD and NVD vulnerability datasets. These datasets cover a wide range of common vulnerability scenarios, providing a reliable benchmark for the experiments. The choice of these datasets is due to their broad coverage of vulnerability types, representing various real-world situations. The sample selection criteria include: coverage of vulnerability types, sample quality, and their representativeness in the industry. The samples extracted from these datasets were filtered based on criteria such as vulnerability type and severity to ensure the diversity and representativeness of the data.

To further validate the applicability and robustness of the proposed method in real-world scenarios, we also introduce the LineVul dataset^32^. This dataset is constructed based on the large-scale Big-Vul dataset and contains a large number of real vulnerability samples extracted from the GitHub open-source code repository. It covers 91 common Common Weakness Enumeration (CWE) types, making it highly representative and of practical value. The sample selection criteria for this dataset include: the types of vulnerabilities, the diversity of vulnerability types, and the complexity of the code.

To ensure the scientific rigor and fairness of the dataset, we conducted systematic preprocessing. The specific steps include:

- Duplicate removal: ensuring that duplicate samples in the dataset are eliminated.
- Code normalization: standardizing the coding format, variable naming, and code style to eliminate unnecessary noise and variation.
- Invalid sample filtering: removing irrelevant or incorrectly labeled samples to ensure the quality of the dataset.
- Label consistency check: ensuring that the labels of all vulnerability samples are accurate and consistent, to avoid training issues caused by labeling errors.

In addition, to ensure the fairness of data partitioning and the reproducibility of the experiments, the dataset was stratified and split in an 80%–10%–10% ratio for training, hyperparameter tuning, and final evaluation. This partitioning protocol ensures that the sample proportions in each subset are consistent, and the distribution of different categories (defective and non-defective) is maintained across the subsets. The four vulnerability types used in the experiments include: library/API function calls (FC), array usage (AU), pointer usage (PU), and arithmetic expressions (AE)^10^. These vulnerability types are all sourced from SySeVR and represent common defect types in code. Ultimately, the generated experimental dataset is diverse and representative, with detailed statistical information provided in Table 1.

**Table 1:** Statistics of vulnerability and non vulnerability samples.

Dataset	Vulnerable type	Vulnerable samples	Non-vulnerable samples	Total
SARD & NVD	AU	8406	46,255	54,661
	PU	20,923	178,778	199,701
	FC	21,590	89,270	110,860
	AE	1853	3332	5185
	Mixed	52,772	317,635	370,407
Big-Vul	AU	1279	6972	8251
	PU	19,555	223,169	242,724
	FC	8821	60,238	69,059
	AE	5521	66,105	71,626
	Mixed	35,176	356,484	391,660

### Experimental environment

All computational experiments utilized NVIDIA A800 GPUs equipped with 80GB graphics memory. Complete hardware specifications and configuration parameters are documented in Table 2.

**Table 2:** Software and environment version information table.

Environment	Version
Operating system	Ubuntu 20.04
CUDA	11.7
Pytorch	2.0.1
Python	3.10
Transformers	4.27.4
Tensorflow	2.7.0
Scikit-learn	1.1.3
Joern	0.3.1
Gensim	4.3.2

### Experimental parameters

The detailed information of the primary hyperparameters for the model in this paper is shown in Table 3.

**Table 3:** Detailed information on model hyperparameters table.

Parameter name	Parameter value
Epochs	20
Batch_size	16
Learning rate	2e-5
Dropout	0.1
Alpha	0.7
Gamma	2.0

The parameter settings of the model involved in this paper are shown in Table 4.

**Table 4:** Model parameter settings table.

Model	Parameter	Value
Tokenizer	vocab_size	50265
	tokenizer_max_length	512
Feature mapping	word_embeddings_sizes	(50265, 768)
	position_embeddings_sizes	(514, 768)
	token_type_embeddings_sizes	(1, 768)
	Dropout	0.1
	hidden_layers_size	(768, 768)
	num_hidden_layers	12
	num_attention_heads	12
	intermediate_size	3072
Classifier	num_hidden_layers	2
	num_hidden_layers_sizes	(768, 768)
	activation_function	Softmax
	loss_function	CrossEntropyLoss + focal_loss
	Dropout	0.1
	num_labels	2

### Experimental results and analysis

To evaluate the effectiveness of the proposed method for code vulnerability detection based on the AUG-PDG and optimized CodeBERT, this paper investigates the following four research questions:

- Experiment 1: Can the proposed method be applied to multiple types of vulnerabilities?
- Experiment 2: Does the proposed method exhibit superior vulnerability detection performance compared to other classical approaches?
- Experiment 3: Does the AUG-PDG proposed in this paper demonstrate better vulnerability detection performance compared to the original PDG?
- Experiment 4: Does the optimized pretrained model proposed in this paper outperform other neural network models in terms of performance?

Experiment 1: Can the proposed method be applied to multiple types of vulnerabilities?

**Fig. 4:**
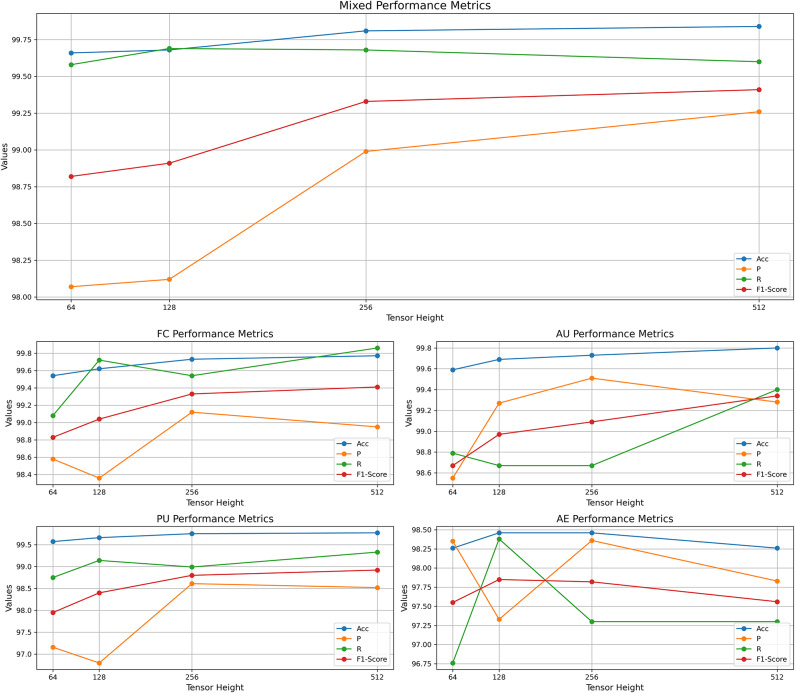
Experimental results of multiple vulnerabilities across different dimensions on the SARD & NVD datasets.

Experiment 1 evaluated the applicability of the proposed method to different vulnerability types by comparing single-vulnerability datasets and multi-vulnerability mixed datasets on the SARD and NVD datasets. In addition, this experiment also explored the impact of the 2D tensor height after code embedding on the experimental results, testing the performance of 64, 128, 256, and 512 as candidate tensor heights. The experimental results are shown in Figure 4.

This experiment focuses on exploring the applicability of the proposed method to different vulnerability types, while also deeply analyzing the impact of tensor height on the model’s performance.

The experimental results indicate that the proposed method performs well across various vulnerability types. As the tensor height increases from 64 to 512, accuracy, precision, recall, and F1 score generally improve, with particularly significant performance on the mixed dataset. When the tensor height is 512, the mixed dataset achieved an accuracy of 99.84%, precision of 99.26%, recall of 99.60%, and an F1 score of 99.41%, demonstrating the model’s excellent performance when integrating multiple vulnerability types.However, for the AE dataset, although there is some fluctuation in performance at different tensor heights, the overall performance remains at a high level. This fluctuation is mainly due to the relatively small sample size of the AE dataset, which results in less stable model training in some cases. Nevertheless, despite these fluctuations, the precision and recall of the AE dataset still indicate the method’s adaptability to smaller datasets, maintaining a certain level of effectiveness and reliability even with fewer samples.

In summary, the experimental results fully demonstrate the applicability and effectiveness of the proposed method in diverse vulnerability detection scenarios, especially with the adjustment of tensor height, where the method exhibits strong flexibility and broad adaptability. Through a comprehensive analysis of different vulnerability types and tensor heights, the proposed method demonstrates high efficiency and stability across various scenarios.

Experiment 2: Does the proposed method exhibit superior vulnerability detection performance compared to other classical approaches?

In Experiment 2, the proposed vulnerability detection method, AUGSliceVul, is compared with several existing advanced and classical methods, including SySeVR, VulDeePecker, Devign, VulCNN, and TRACED. Below is a brief introduction to these five baseline methods:

- VulDeePecker^7^: Uses Bi-LSTM to process semantically related code lines, serializing them into vectors for model training.
- SySeVR^10^: Reduces noise in the samples through program slicing and uses Bi-LSTM to process the denoised data, thereby enhancing the model’s ability to accurately learn vulnerability features.
- Devign^8^: Constructs a composite code graph and applies Graph Neural Networks (GNNs) to capture semantic information embedded in the graph, leveraging GNN to understand code structure and semantic characteristics.
- VulCNN^11^: Uses Program Dependency Graphs (PDGs) as the intermediate representation of the program and utilizes Convolutional Neural Networks (CNN) to extract semantic information from these graphs.
- TRACED^33^: An execution-aware pretraining framework for source code. Its core is to combine program dynamic execution traces with static code structures, optimizing code representations through bimodal contrastive learning.

**Fig. 5:**
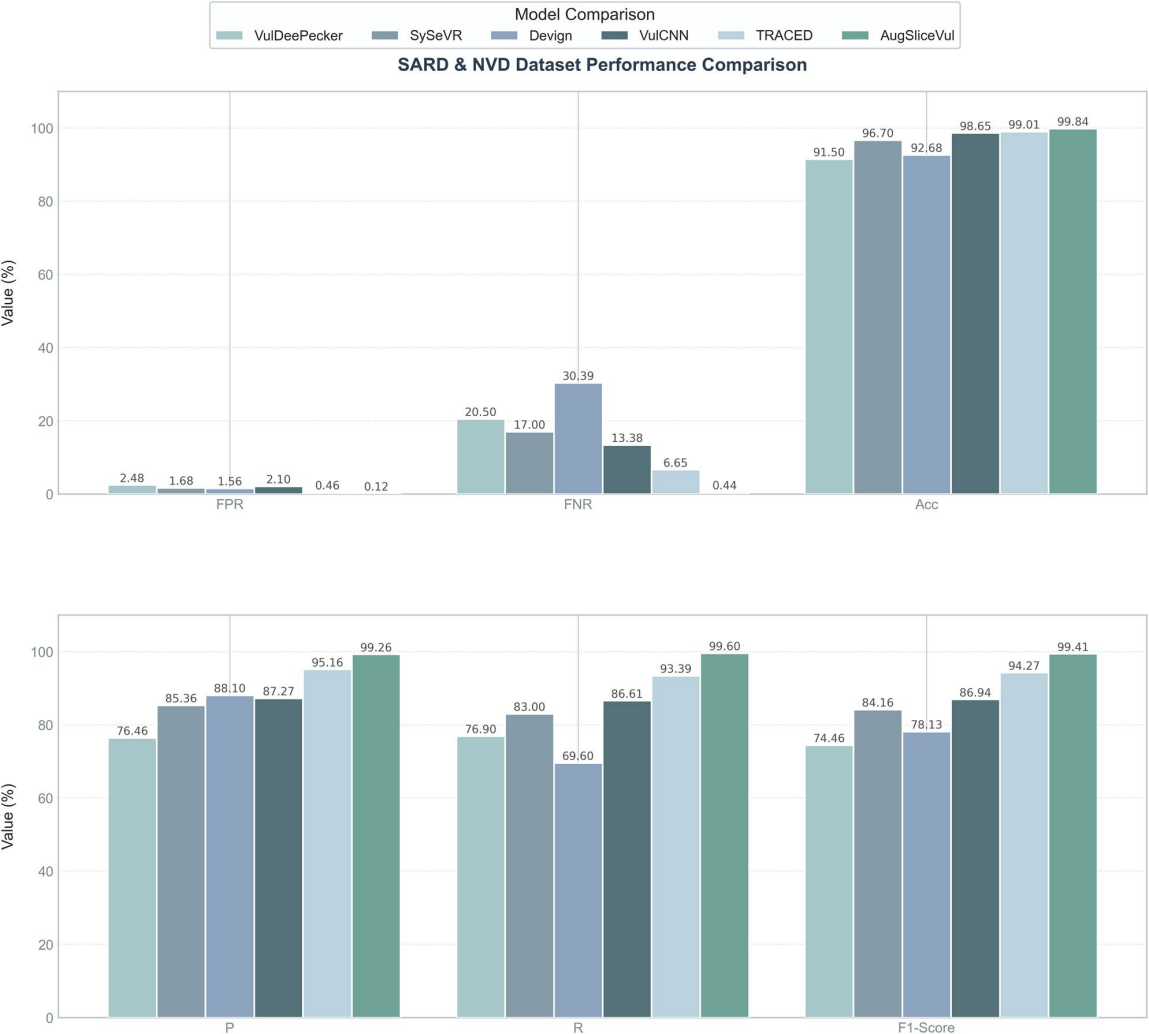
Experimental comparison results of different methods (SARD & NVD dataset).

**Fig. 6:**
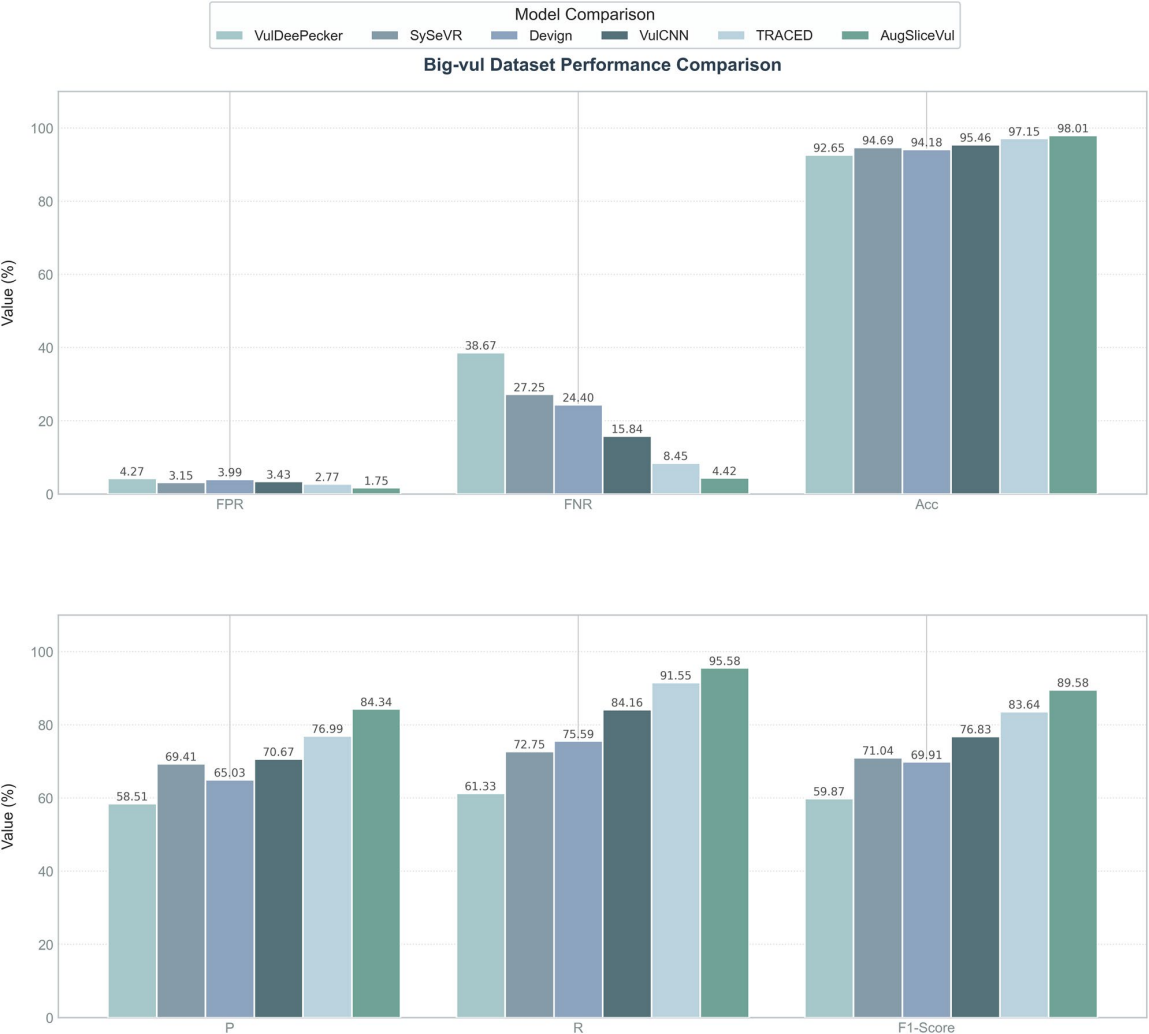
Experimental comparison results of different methods(Big-Vul dataset).

Figures 5 and 6 display the experimental results of these models on the SARD & NVD dataset and the Big-Vul dataset, respectively.

The experimental results show that AUGSliceVul significantly outperforms existing methods across multiple performance metrics, fully validating its effectiveness and applicability in vulnerability detection tasks. Specifically, on the SARD & NVD dataset, AUGSliceVul achieved an average accuracy of 99.84%, precision of 99.26%, recall of 99.60%, and F1 score of 99.41%. Compared to other models, the F1 score improved by 5.14–24.95%, recall by 6.21–30.00%, precision by 4.1–22.80%, and accuracy by 0.83–8.34%. The false negative rate and false positive rate were only 0.44% and 0.12%, respectively, which are more than ten times lower than other models. On the Big-Vul dataset, AUGSliceVul also achieved an average accuracy of 98.01%, precision of 84.34%, recall of 95.58%, and F1 score of 89.58%. Compared to TRACED, accuracy improved by 0.86%, precision by 7.35%, recall by 4.03%, and F1 score by 5.94%. Compared to other models, the F1 score improved by 12.75–29.71%, recall by 11.42–34.25%, precision by 13.76–25.83%, and accuracy by 2.55%–5.36%. The false negative rate and false positive rate were only 4.42% and 1.75%, respectively, significantly lower than those of other methods, especially on large-scale datasets, where AUGSliceVul consistently maintained low false positive and false negative rates.

To further validate the significance of the experimental results, we performed an analysis of variance (ANOVA) on the performance of six models (AUGSliceVul, VulDeePecker, SySeVR, Devign, VulCNN, and TRACED) on the SARD & NVD dataset. First, the data were validated for normality using the Shapiro-Wilk test (all groups p > 0.05) and for homogeneity of variance using Levene’s test (p = 0.784), indicating that the data met the assumptions of normality and homogeneity of variance. Next, a one-way ANOVA was conducted, and the results showed that there were highly significant differences in the F1 scores among the six models (F(5,24) = 8503.29, p < 0.001, $\eta ^2$ = 0.999). The effect size $\eta ^{2}$ = 0.999 indicates that the model type nearly fully explains the variance in F1 scores, demonstrating significant performance differences among the models. To further analyze the differences between models, a Tukey HSD post hoc test was conducted, and the results are shown in Table 5. Among them, the mean F1 score of AUGSliceVul was significantly higher than the other models, with an improvement of 5.09% over the second-ranked TRACED, and 25.11% and 21.02% higher than the traditional models VulDeePecker and Devign, respectively. The performance of the six models displayed a strict hierarchical order: AUGSliceVul > TRACED > VulCNN > SySeVR > Devign > VulDeePecker, with significant differences between adjacent groups (e.g., TRACED vs VulCNN with a difference of 7.58%, VulCNN vs SySeVR with a difference of 2.49%). The statistical analysis results indicate that AUGSliceVul outperforms existing vulnerability detection models, and its statistical significance and practical applicability are fully validated.

**Table 5:** Tukey HSD post inspection results.

Group1	Group2	Meandiff	95% confidence interval	p-adj	Reject
AUGSliceVul	Devign	+21.02	[20.58, 21.47]	< 0.001	True
AUGSliceVul	SySeVR	+15.16	[14.71, 15.61]	< 0.001	True
AUGSliceVul	TRACED	+5.09	[4.64, 5.54]	< 0.001	True
AUGSliceVul	VulCNN	+12.67	[12.22, 13.11]	< 0.001	True
AUGSliceVul	VulDeePecker	+25.11	[24.66, 25.56]	< 0.001	True
Devign	SySeVR	-5.87	[5.42, 6.31]	< 0.001	True
Devign	TRACED	-15.94	[15.49, 16.39]	< 0.001	True
Devign	VulCNN	-8.36	[7.91, 8.81]	< 0.001	True
Devign	VulDeePecker	+4.08	[3.63, 4.53]	< 0.001	True
SySeVR	TRACED	-10.07	[9.62, 10.52]	< 0.001	True
SySeVR	VulCNN	-2.49	[2.04, 2.94]	< 0.001	True
SySeVR	VulDeePecker	+9.95	[9.50, 10.40]	< 0.001	True
TRACED	VulCNN	+7.58	[7.13, 8.03]	< 0.001	True
TRACED	VulDeePecker	+20.02	[19.57, 20.47]	< 0.001	True
VulCNN	VulDeePecker	+12.44	[11.99, 12.89]	< 0.001	True

In summary, AUGSliceVul has demonstrated significant performance improvements across various vulnerability detection tasks. Through one-way ANOVA and Tukey HSD post hoc tests, the relative advantage of this method among different models has been validated, proving its broad applicability and efficiency in practical applications. The comprehensive improvement in key metrics such as accuracy, precision, recall, and F1 score highlights the method’s performance and versatility.

Experiment 3: Does the AUG-PDG proposed in this paper demonstrate better vulnerability detection performance compared to the original PDG?

This paper conducts a comparative analysis between AUG-PDG and traditional PDG across multiple models, evaluating their code representation efficacy through vulnerability detection performance. The experiment selected three commonly used neural network architectures–RNN, LSTM, and BiLSTM–along with the modified CodeBERT model proposed in this paper, referred to as Opt_CodeBERT to distinguish it from the original CodeBERT model. All architectures were benchmarked with both baseline PDG and AUG-PDG for code representation. Empirical validation utilized the SARD & NVD dataset and Big-Vul dataset in separate trials. A detailed summary of the experimental results can be found in Tables 6 and 7.

**Table 6:** Comparison of vulnerability detection performance using different program representation methods on the SARD & NVD datasets (unit: %).

		FNR	Acc	P	R	F1-score
RNN	PDG	95.88	88.76	52.10	4.12	7.63
	AUG-PDG	**56.77**	**90.51**	**73.66**	**43.12**	**54.39**
LSTM	PDG	19.40	96.77	88.35	80.60	84.30
	AUG-PDG	**12.54**	**97.10**	**91.43**	**87.46**	**89.40**
BILSTM	PDG	18.66	96.37	83.80	81.34	82.55
	AUG-PDG	**7.87**	**97.74**	**91.50**	**92.13**	**91.82**
Opt_CodeBERT	PDG	1.46	99.57	97.49	98.55	98.01
	AUG-PDG	**0.44**	**99.84**	**99.26**	**99.60**	**99.41**

Better-performing indicator values are in bold.

**Table 7:** Comparison of vulnerability detection performance using different program representation methods on the Big_vul datasets (unit: %).

		FNR	Acc	P	R	F1-score
RNN	PDG	82.27	**94.62**	**97.29**	17.73	29.99
	AUG-PDG	**72.08**	93.84	96.86	**27.92**	**43.34**
LSTM	PDG	53.65	96.01	86.17	46.35	60.27
	AUG-PDG	**36.56**	**96.07**	**88.86**	**63.44**	**74.03**
BILSTM	PDG	70.01	94.86	77.39	29.99	43.23
	AUG-PDG	**42.74**	**95.81**	**91.81**	**57.26**	**70.54**
Opt_CodeBERT	PDG	25.16	97.47	**86.40**	74.84	80.24
	AUG-PDG	**4.42**	**98.01**	84.34	**95.58**	**89.58**

Better-performing indicator values are in bold.

The experimental results indicate that code slices based on the AUG-PDG representation outperform those based on the original PDG representation across all models, particularly excelling in key evaluation metrics.

Specifically, first, AUG-PDG significantly reduced the false negative rate (FNR) across all models. Taking the RNN model as an example, under the PDG representation, the FNR was as high as 95.88%, while after using AUG-PDG, the FNR decreased to 56.77%, a reduction of nearly 40%. This indicates that AUG-PDG significantly improved the model’s sensitivity to vulnerability features, reducing the occurrence of false negatives. On the Big-Vul dataset, the RNN model’s FNR decreased from 82.27% to 72.08%, further verifying the significant enhancement in vulnerability detection ability brought by AUG-PDG.

Second, while improving detection performance, AUG-PDG also led to improvements in accuracy (Acc) and F1 score. On the SARD & NVD dataset, the F1 score of the RNN model increased from 7.63% to 54.39%, a rise of 46.76%; the F1 scores of the LSTM and BiLSTM models increased from 84.30% and 82.55% to 89.40% and 91.82%, respectively. On the Big-Vul dataset, the F1 score of the LSTM model increased from 60.27% to 74.03%, and the BiLSTM model’s F1 score increased from 43.23% to 70.54%. These improvements demonstrate that AUG-PDG, by incorporating more syntactic and semantic information, helps the model better recognize potential vulnerability features, thereby improving the accuracy of feature representation.

In addition, AUG-PDG also showed significant improvement in balancing precision and recall. Taking the Big-Vul dataset as an example, AUG-PDG improved the recall rate in the LSTM and BiLSTM models from 46.35% and 29.99% to 63.44% and 57.26%, respectively, significantly enhancing recall ability and reducing missed vulnerabilities. Through the enhanced program dependency graph, AUG-PDG is able to capture vulnerability patterns in the code more comprehensively, making the model more sensitive when identifying vulnerabilities, further improving overall performance.

Finally, Opt_CodeBERT demonstrated the best performance on both the SARD & NVD and Big-Vul datasets. On the SARD & NVD dataset, the F1 score of Opt_CodeBERT reached 99.41%, an improvement of 1.40% compared to the PDG-based Opt_CodeBERT (98.01%); on the Big-Vul dataset, the F1 score increased from 80.24% to 89.58%, showing the further enhancement effect of AUG-PDG in efficient encoding models.

The experimental results fully demonstrate the effectiveness of AUG-PDG as an enhanced code representation. By introducing additional syntactic and semantic relationship edges, AUG-PDG enables the model to better understand potential vulnerabilities in the code. Compared to traditional Program Dependency Graphs (PDG), AUG-PDG shows stronger vulnerability detection capabilities across multiple metrics, particularly in the significant reduction of false negative rates and the improvement of accuracy, precision, recall, and F1 score. The effectiveness of AUG-PDG is explained in detail in Section Augmented program dependency graph of this paper, and its superiority is verified through experiments presented here.

Experiment 4: Does the optimized pretrained model proposed in this paper outperform other neural network models in terms of performance?

**Fig. 7:**
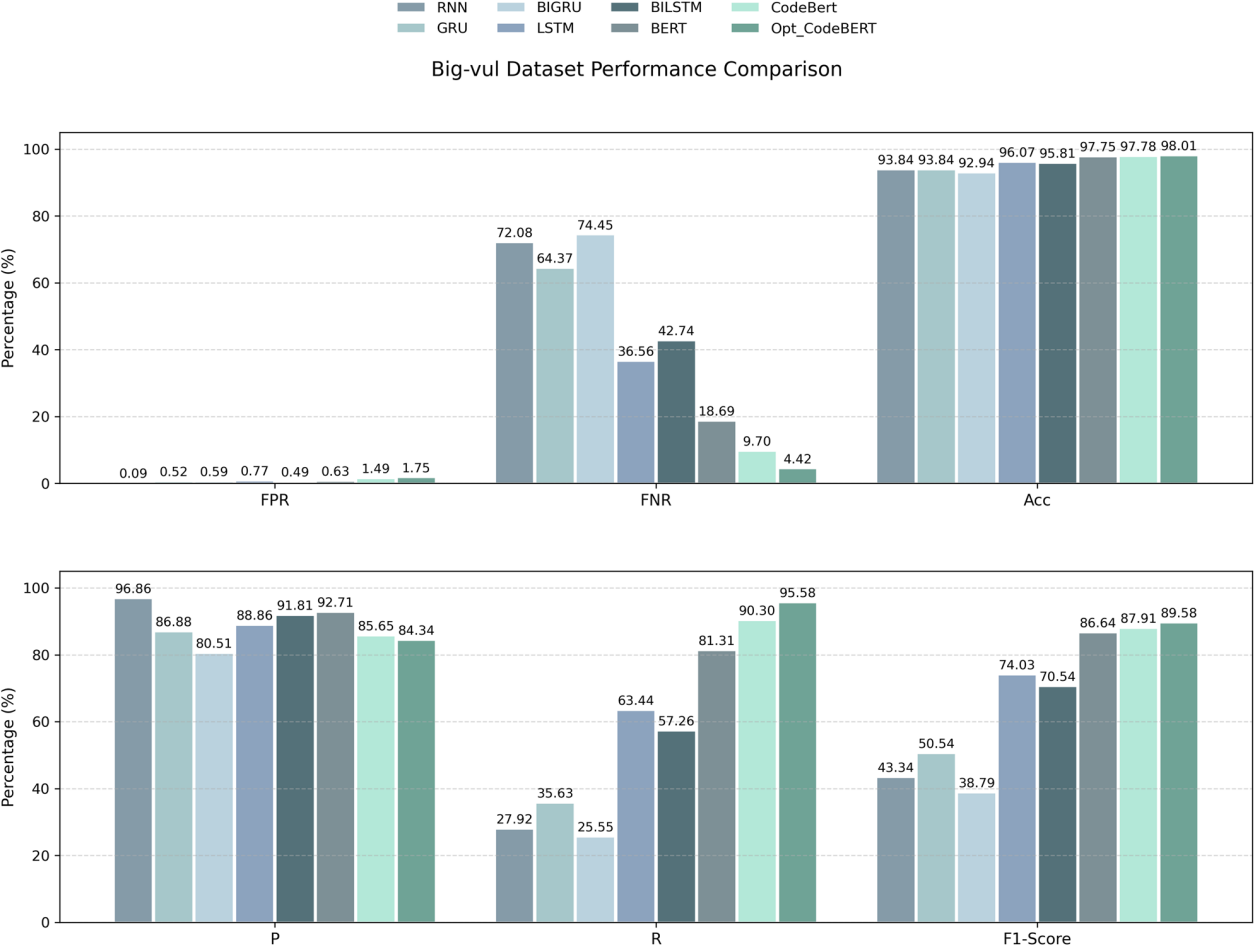
Performance comparison of different models in vulnerability detection tasks using AUG-PDG slice datasets.

To comprehensively evaluate the performance of the improved pretrained model proposed in this paper, seven representative models were selected for comparison, including including RNN, LSTM, GRU, BiLSTM, BiGRU, the BERT pretrained model and the CodeBERT pretrained model. All models were trained and tested under identical experimental settings and on the same dataset to ensure fairness and comparability of the results. The detailed outcomes are presented in Fig.  7.

The experimental results show that Opt_CodeBERT outperforms other models in terms of false negative rate (FNR) and recall (R), with a false negative rate of only 4.42% and a recall rate of 95.58%. This significant advantage indicates that Opt_CodeBERT not only captures vulnerability features more comprehensively but also effectively reduces the missed detection rate, enhancing the overall sensitivity of detection. In contrast, traditional models such as RNN, LSTM, and BiLSTM, while having higher accuracy, often fail to fully identify potential vulnerability features, leading to higher false negative rates and lower recall rates. Moreover, although Opt_CodeBERT’s false positive rate (FPR) is slightly higher than some traditional models, this gap does not affect its overall performance. Traditional models have fewer false positives due to weaker vulnerability detection capabilities, but their high accuracy comes at the cost of lower recall rates. Opt_CodeBERT, through enhanced code representation, successfully balances precision and recall, significantly improving recall while keeping the false positive rate within an acceptable range. This makes Opt_CodeBERT more valuable in practice when handling multiple types of vulnerabilities. In terms of F1 score, Opt_CodeBERT also performs excellently, achieving 89.58%. This result reflects the model’s success in balancing precision and recall, and the improvement in F1 score, especially in multi-type vulnerability detection tasks, proves its strong capability in multi-dimensional vulnerability detection. Compared with traditional models, Opt_CodeBERT, with its pretraining advantages and enhanced code representation methods, is better at understanding the deep structure of code, effectively avoiding the common shortcomings of traditional models in vulnerability recognition, significantly improving the control of false negative rates and enhancing overall performance.

In summary, Opt_CodeBERT demonstrates significant advantages across multiple evaluation metrics, particularly in false negative rate, recall, and F1 score. Although it performs slightly worse than some traditional models in terms of accuracy and false positive rate, its strength lies in its high recall and extremely low false negative rate, allowing it to provide more comprehensive and accurate vulnerability detection results in practical applications. The Opt_CodeBERT model optimizes the CodeBERT classification model loss through a hybrid loss function optimization strategy, addressing the long-tail distribution characteristics of code vulnerability detection.

## Conclusion

Addressing the limitations of conventional static analysis approaches and neural detection systems in terms of detection granularity and information loss, this paper proposes a code vulnerability detection method based on the AUG-PDG and an optimized CodeBERT. To enhance the expression of code semantics and structure, this paper proposes the construction of an AUG-PDG to extend the traditional program dependence graph (PDG). By applying a dynamic root node selection strategy and a bidirectional dependency traversal mechanism to the program slicing algorithm, noise interference in code vulnerability detection is reduced. CodeBERT is employed as the code embedding model to embed and encode the program slices, extracting their deep structural and semantic features. To address the issue of class imbalance in the samples, this paper introduces a hybrid loss function optimization strategy tailored to CodeBERT, strengthening the model’s ability to recognize sample classes and reducing false positives and false negatives. Experimental results demonstrate that the proposed method achieves improvements of up to 8.34% in accuracy and 29.71% in F1 score compared to other traditional methods. The detection approach presented in this paper offers a technically valuable solution for software vulnerability detection.

However, this method still has certain limitations, while also pointing out directions for future research work: 1) Further refinement of code structure analysis to achieve more granular vulnerability detection at the line or statement level. 2) Investigating model adaptability across different programming languages and paradigms to develop a generalized vulnerability detection framework. 3) Expanding intermediate program representation techniques to incorporate richer semantic and syntactic information relevant to vulnerabilities, thereby enhancing detection accuracy and robustness.

## Data Availability

The dataset used in this study originates from the available dataset provided in the SySeVR framework (https://github.com/SySeVR/SySeVR.git ). Minor adjustments were made to align with the experimental setup of this research, ensuring compatibility with the proposed method. The modified dataset can be accessed at: https://github.com/yunk2000/AUG Slice/tree/main/data sets.The source code for this study, including scripts for data processing and model imple-mentation, is openly available on GitHub: https://github.com/yunk2000/AUG Slice. The repository includes detailed instructions for replicating the experiments and applying the described modifications.
